# Proteogenomics unveils mechanistic insights to precision oncology

**DOI:** 10.1002/ctm2.1477

**Published:** 2023-11-27

**Authors:** Yongchao Dou, Lizabeth Katsnelson, Bing Zhang, David Fenyö, Tao Liu

**Affiliations:** ^1^ Lester and Sue Smith Breast Center Baylor College of Medicine Houston Texas USA; ^2^ Department of Molecular and Human Genetics Baylor College of Medicine Houston Texas USA; ^3^ Dan L Duncan Comprehensive Cancer Center Baylor College of Medicine Houston Texas USA; ^4^ NYU Grossman School of Medicine Institute for Systems Genetics New York New York USA; ^5^ Department of Biochemistry and Molecular Pharmacology NYU Grossman School of Medicine New York New York USA; ^6^ Biological Sciences Division Pacific Northwest National Laboratory Richland Washington USA

**Keywords:** Cancer Biology, Mass Spectrometry, Oncology, Proteogenomics

1

Even though genomics plays a critical role in the development of precision treatments for cancer, the actual treatments overwhelmingly target proteins, which are responsible for most cellular function.^1^ Moreover, from genomic sequencing alone, it is not possible to predict how translation is regulated, the rate of degradation of proteins, and post‐translational modifications (PTMs) that are often independently regulated. Therefore, genomics alone cannot provide comprehensive information for precision treatment. Consequently, the integration of proteomics, transcriptomics and genomics in the field of proteogenomics holds the potential to unveil novel mechanisms and therapeutic targets.^2^ Along this path, the National Cancer Institute's Clinical Proteomic Tumor Analysis Consortium (CPTAC) has carried out proteogenomic characterization for over 10 cancer types.^3^ Many regulatory mechanisms and new therapeutic opportunities were revealed by integrated multi‐omics analysis.

As a part of the CPTAC initiative, we recently published a prospective proteogenomic study focusing on endometrial carcinoma (EC).^4^ In this study, we characterized an independent cohort of 138 prospectively collected EC tumors and 20 enriched normal tissues using 10 different omics platforms (Figure 1). Subsequently, incorporating two previously published EC cohorts,^5^, ^6^ we conducted multi‐omics and multi‐cohort analyses. In the following sections, we present two examples from our study to illustrate how proteogenomics aids in the discovery of druggable pathways and novel regulatory mechanisms.

**FIGURE 1:**
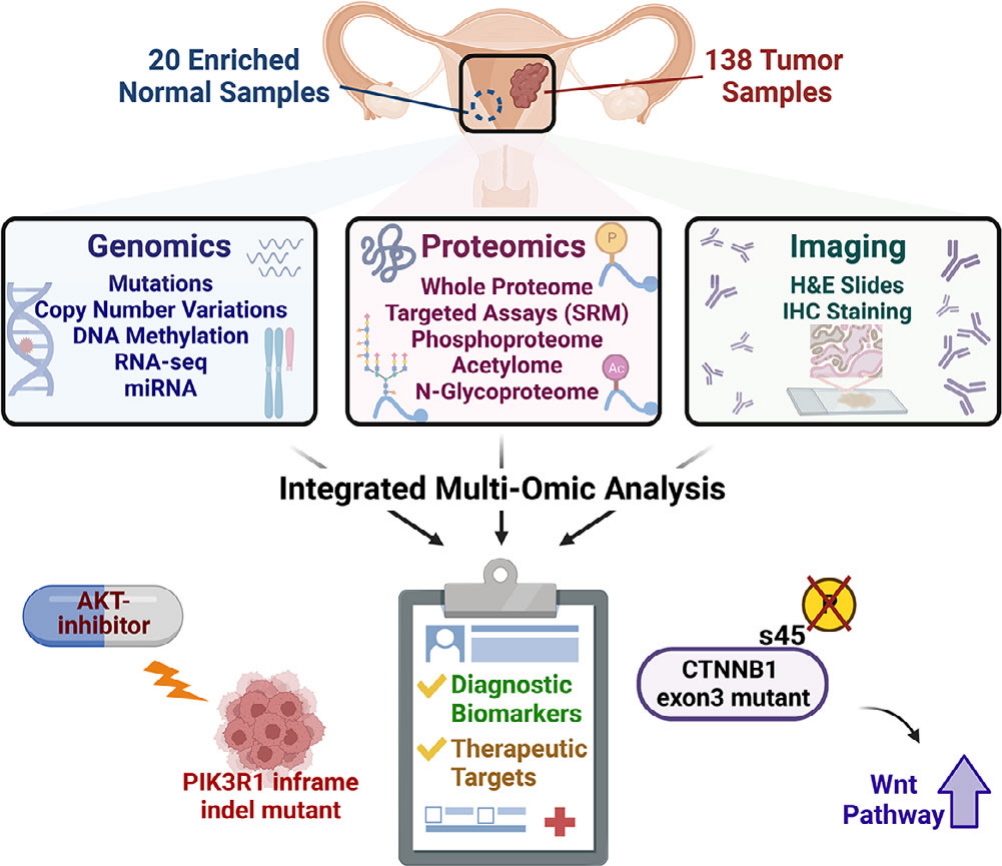
A prospective proteogenomic study of endometrial carcinoma (EC). Our proteogenomic characterization provides several valuable insights, including an association between PIK3R1 in‐frame indels and up‐regulated AKT1 phosphorylation, as well as how CTNNB1 exon 3 hotspot mutations block pS45‐induced β‐catenin degradation (figure created by BioRender).

In the first example, a proteogenomic investigation of genomic variants within the PI3K/AKT pathways unveiled that PIK3R1 in‐frame variants promote PI3K/AKT activity solely at the AKT1 phosphorylation level. As one of the most frequently perturbed pathways in human tumors, many drugs have been developed to target the PI3K/AKT pathway. However, most of them have failed in clinical trials. Notably, the selection of patients for these trials has often relied on the presence of PIK3CA hotspot mutations.^7^ A closer examination reveals that PIK3CA and PIK3R1 mutations are mutually exclusive in EC, and PIK3R1 harbors a much higher proportion of in‐frame variants than other top mutated genes. Interestingly, these in‐frame indels are tightly clustered in a region of PIK3R1 that interacts with PIK3CA. Since PIK3R1 is a suppressor of AKT phosphorylation, truncating mutations are expected to activate the PI3K/AKT pathway and lead to worse clinical outcomes in EC patients. Surprisingly, we did not observe a difference in overall survival in EC patients harboring PIK3R1 truncating or PIK3CA hotspot mutations compared to patients with WT PIK3R1 tumors. In contrast, PIK3R1 infame variants were associated with significantly worse survival than both PIK3R1 truncating and PIK3CA hotspot patients. Moreover, PIK3R1 in‐frame variants were associated with the highest levels of AKT1‐T308 phosphorylation compared with PIK3CA hotspot and PIK3R1 truncating mutations. We confirmed this finding by creating a PIK3R1 in‐frame indel by CRISPR (Clustered Regularly Interspaced Short Palindromic Repeats) in an EC cell line. Analysis of drug response data from DepMap showed that endometrial cell lines with PIK3R1 in‐frame variants were more sensitive to AKT inhibitors, compared to cell lines with PIK3CA hotspot, PIK3R1 truncation, and WT PIK3R1.^8^ In summary, PIK3R1 in‐frame variants may be a better biomarker than PIK3CA hotspot mutations for AKT inhibition.

In the second example, our proteogenomic analysis revealed the mechanisms behind the accumulation of β‐catenin protein and resistance to Wnt/FZD antagonists in CTNNB1 hotspot‐mutated tumors, achieved through the dephosphorylation of β‐catenin at S45. Hotspot mutations within exon 3 of CTNNB1 result in a significant upregulation of the WNT/β‐catenin pathway and are associated with poorer survival and recurrence outcomes. Consequently, many drugs have been designed to target upstream regulators of β‐catenin, including DKK and FZD. These drugs function by releasing the β‐catenin phosphorylation complex which facilitates the phosphorylation of β‐catenin. Subsequently, the phosphorylated β‐catenin undergoes degradation. While the most significantly up‐regulated protein in CTNNB1 hotspot mutated tumors is DKK4, an inhibitor of the WNT pathway,^9^ CTNNB1 and its downstream LEF1 are still significantly up‐regulated at the protein level, despite elevated DKK. In‐depth investigations unveil that CTNNB1 hotspot mutations target key phosphorylation sites S33, S37, T41, and S45, and their neighboring sites D32 and G34. These mutations may block phosphorylation‐dependent binding of ubiquitin ligases and subsequent 𝛃‐catenin degradation.^10^ Consistent with this hypothesis, S45 phosphorylation was significantly lower in samples with hotspot mutations. All these results suggest that CTNNB1 hotspot mutations blocked pS45 induced 𝛃‐catenin degradation. Thus, new strategies are needed to target 𝛃‐catenin and inhibit the WNT pathway.

Additional findings include: (1) A negative correlation was observed between MYC activity and treatment with the anti‐diabetic drug metformin, which has been shown to improve overall survival of diabetic EC patients. As many non‐diabetic patients displayed elevated MYC activity, these results suggest a role for metformin treatment in non‐diabetic EC patients. (2) Targeted Selected Reaction Monitoring analysis identified two peptides that can accurately predict activity of the antigen processing and presentation machinery, providing candidate biomarkers to inform selection of EC patients for treatment with immune checkpoint inhibitors. (3) EC tumors with gain of chromosome 1q have lower immune activity and decreased disease‐free survival. EC cell lines in DepMap with PARP1 amplification are more sensitive to PARP inhibition. Based on these findings, PARP1 gain is a potential biomarker for PARP inhibitor therapy of EC. (4) Non‐negative matrix factorization clustering of glycopeptides groups tumors into four subtypes that are distinct from the multi‐omics clusters. (5) Deep learning models accurately predicted EC subtypes and mutations from analysis of histopathology images, which may be useful for rapid diagnosis of certain subtypes as a complement of genome sequencing.

In summary, this study identified molecular markers and mechanisms that can be further investigated to guide patient stratification for precision treatment of EC. Notably, proteomics and phosphoproteomics played a key role in the path to these findings. Furthermore, these findings underscore the promise of proteogenomics, an integration of proteomics, transcriptomics, and genomics, for providing profound insights in precision oncology.

## CONFLICT OF INTEREST STATEMENT

The authors declare no conflicts of interest.
